# Enhanced Phenotype Identification of Common Ocular Diseases in Real-World Datasets

**DOI:** 10.1016/j.xops.2025.100717

**Published:** 2025-01-24

**Authors:** Joshua D. Stein, Hong Su An, Chris A. Andrews, Suzann Pershing, Tushar Mungle, Amanda K. Bicket, Julie M. Rosenthal, Amy D. Zhang, Wen-Shin Lee, Cassie Ludwig, Bethlehem Mekonnen, Tina Hernandez-Boussard, SOURCE consortium, Sejal Amin, Paul A. Edwards, Divya Srikumaran, Fasika Woreta, Jeffrey S. Schultz, Anurag Shrivastava, Louis R. Pasquale, Baseer Ahmad, Paul Bryar, Dustin D. French, Michelle Hribar, Merina Thomas, Rajeev Ramachandran, Brian L. Vanderbeek, Suzann Pershing, Sophia Y. Wang, Michael Deiner, Catherine Sun, Jennifer Patnaik, Prem Subramanian, Saleha Munir, Wuqaas Munir, Joshua D. Stein, Lindsey De Lott, Robert Feldman, Brian C. Stagg, Barbara Wirostko, Brian McMillian, Arsham Sheybani, Ji Liu, Soshian Sarrapour

**Affiliations:** 1 Department of Ophthalmology and Visual Sciences, University of Michigan, Ann Arbor, Michigan; 2 Department of Health Management and Policy, School of Public Health, University of Michigan, Ann Arbor, Michigan; 3 Department of Ophthalmology and Visual Sciences, Byers Eye Institute, Stanford University, Palo Alto, California; 4 Department of Medicine, Stanford University, Palo Alto, California

**Keywords:** Electronic health records, Machine learning, Diabetic retinopathy, Macular degeneration, Glaucoma

## Abstract

**Objective:**

For studies using real-world data, accurately identifying patients with phenotypes of interest is challenging. To identify cohorts of interest, most studies exclusively use the International Classification of Diseases (ICD) billing codes, which can be limiting. We developed a method to accurately identify the presence or absence of 3 common ocular diseases (diabetic retinopathy [DR], age-related macular degeneration [AMD], and glaucoma) using electronic health record (EHR) data.

**Design:**

Database study.

**Participants:**

Three thousand nine hundred fourteen eyes from 1957 patients at 2 Sight OUtcomes Research CollaborativE (SOURCE) Ophthalmology Data Repository sites.

**Methods:**

We developed enhanced phenotype identification (EPI) algorithms that search EHR fields, including eye examination findings, orders, charges, medication prescriptions, and surgery data for evidence that a patient has glaucoma, DR, or AMD. We trained our EPI models using gold standard assessments of the EHR by ophthalmologists for the presence/absence of these conditions, compared the performance of our EPI models to models developed using ICD codes alone, and validated the performance of model using data from another SOURCE site.

**Main Outcome Measures:**

Area under the receiver operating curve (AUC), area under the precision–recall curve (AUPRC), and model calibration.

**Results:**

The AUCs of our EPI models were better than ICD-only models for glaucoma (0.97 vs. 0.90), DR (0.997 vs. 0.98), and AMD (0.99 vs. 0.95). The AUPRCs of our EPI models were also much better than ICD-only models for glaucoma (0.79 vs. 0.32), DR (0.96 vs. 0.84), and AMD (0.74 vs. 0.55). When testing on patients from a second SOURCE site, the AUC and AUPRC for glaucoma (0.93, 0.74), DR (0.98, 0.77), and AMD (0.96, 0.64) were slightly worse than the primary site but still quite high. However, for all 3 conditions, model calibration was worse at the second site.

**Conclusions:**

Leveraging machine learning, we developed EPI models to accurately identify most patients with glaucoma, DR, and AMD in real-world datasets. The EPI models significantly outperform ICD-only models in identifying patients confirmed to have these conditions. These findings underscore the potential of using comprehensive EHR data combined with advanced machine learning techniques to improve the accuracy of patient phenotype identification, leading to better patient management and clinical outcomes.

**Financial Disclosure(s):**

Proprietary or commercial disclosure may be found in the Footnotes and Disclosures at the end of this article.

Researchers have used real-world data to study the epidemiology of ocular diseases,^1^^,^^2^ the natural history and burden of disease,^3^, ^4^, ^5^ treatment patterns and outcomes,^6^^,^^7^ and eye care disparities.^8^^,^^9^ Most such studies used International Classification of Disease (ICD) billing codes to identify patients with the conditions of interest. Patients with a code for a particular ocular disease typically have corroborating diagnostic evidence elsewhere in the electronic health record (EHR); meanwhile, billing codes may be incomplete, inaccurate, or insufficiently granular to distinguish between different conditions.^10^ In 1 study in which pseudoexfoliation was indicated somewhere in the EHR, only 12% to 26% of visits received an ICD-9/10 billing code for that diagnosis.^11^

In health care analyses using only claims data, billing codes are the main means to identify which patients have particular diseases. As researchers shift away from reliance on claims data, data from various EHR sections may supplement billing code information.^12^ Although using ICD codes may suffice for answering some research questions, others may require a sensitivity and specificity unattainable with billing codes alone.^13^

We developed enhanced phenotype identification (EPI), an approach that leverages machine learning and incorporates data from multiple EHR areas to identify patients with and without ocular diseases of interest. This approach performed well previously in differentiating patients with and without pseudoexfoliation.^11^ The current study aims to develop and validate EPI algorithms for 3 common sight-threatening diseases: glaucoma, diabetic retinopathy (DR), and age-related macular degeneration (AMD). We compare the accuracy and other performance metrics of ICD-only models with our EPI models, hypothesizing that the latter can more accurately identify the presence or absence of these common ocular diseases. Improving the accuracy of disease identification through enhanced methods could significantly elevate the quality of research involving real-world data, leading to more reliable epidemiological analyses, better understanding of disease progression, and improved evaluation of treatment outcomes.

## Methods

### Data Source

We used EHR data on patients receiving any eye care at the University of Michigan (“Michigan”), 2012 to 2021, or Stanford University (“Stanford”), 2013 to 2021, 2 member sites of the Sight OUtcomes Research CollaborativE (SOURCE). All SOURCE sites use EPIC EHR software. The SOURCE data were used previously to study ocular diseases^14^^,^^15^ (for more details on SOURCE, see www.sourcecollaborative.org). Michigan’s and Stanford’s Institutional Review Boards approved this study, and our study adheres to the Declaration of Helsinki.

### Variables Considered in the EPI Models

We searched these EHR locations for evidence indicating the presence or absence of glaucoma, DR, and AMD:

1. Billing data: ICD-9/10 codes for glaucoma (ICD-9: 365.X; ICD-10: H40.X), DR (ICD-9: 362.0x; ICD-10: E10.31-E10.35, E11.31-E11.350), and AMD (ICD-9: 362.51, 362.50, 362.52, 362.57; ICD-10: H35.31, H35.36, H35.32, H35.30);
2. Sociodemographic characteristics of the patient: age, sex, race, ethnicity, marital status, employment status, health insurance type;
3. Problem list: a running list of patients’ medical conditions during their time in the health system;
4. Clinical examination: using regular expressions and accounting for negation, as described elsewhere,^11^ we searched semistructured data in clinic notes for ocular examination findings indicating evidence of glaucoma, DR, and AMD in designated fields for conjunctiva, cornea, anterior chamber, iris, lens, optic nerve, cup-to-disc, vitreous, macula, vessels, and periphery;
5. Orders and charges: for diagnostic testing (e.g., perimetry, retinal or optic nerve OCT, fundus photography) or therapeutic procedures (e.g., glaucoma lasers, intravitreal injections);
6. Outpatient medications: prescribed topical glaucoma medications; oral hypoglycemics for diabetes;
7. Outpatient labs: glycated hemoglobin;
8. Surgery data: Current Procedural Terminology codes for glaucoma, DR, or AMD surgical and procedural interventions.

In total, 216 unique variables were considered (Table 1 and Table S2, available at www.ophthalmologyscience.org).

**Table 1:** Variables Captured in the Electronic Health Record Considered to Predict the Presence of Glaucoma, Diabetic Retinopathy, and Macular Degeneration

Category	Examples of Variables
Sociodemographics	Age, sex, race, ethnicity, marital status, employment status, health insurance type
Diagnoses	Systemic medical conditions such as diabetes, hypertension, and depression. Charlson comorbidity index,∗ alcohol use, tobacco use, problem list entries
Clinical examination and findings	Findings from semistructured data captured in eye examination fields such as pupils, conjunctiva, cornea, iris, anterior chamber, lens, vitreous, optic nerve, cup-to-disc, macula, vessels, periphery, and intraocular pressure
Orders and charges	Pachymetry, indocyanine green angiography, intravenous fluorescein angiography, anti-VEGF injections, OCT of the macula, OCT of the optic nerve, perimetry
Outpatient medications	Glaucoma medications including alpha agonists, beta blockers, carbonic anhydrase inhibitors, prostaglandin analogs, rho kinase inhibitors, and parasympathomimetics. Diabetes medications include sulfonylureas, meglitinides, biguanides, thiazolidinediones, alpha-glucoside inhibitors, SGLT2 inhibitors, glucagon-like peptide (GLP-1) receptor agonists, and insulin.
Outpatient labs	Glycated hemoglobin
Surgery	CPT-4 codes for different glaucoma surgeries: 0191T, 0253T, 0449T, 0450T, 0671T, 65820, 65850, 65855, 66170, 66172, 66174, 66175, 66179, 66180, 66183, 66184, 66185, 66710, 66711, 66761, 66762, 66987, 66988, 66989, 66991. Retinal surgeries/procedures: 67028, C9093, C9142, C9291, C9399, J0178, J0179, J2778, J2779, J3490, J3590 J7999, J9035, Q2046, Q5107, Q5107, Q5118, Q5124, 67221, 67225.

CPT = Current Procedural Terminology; SGLT2 = sodium–glucose transport protein 2.

In total, the models considered 216 unique variables.

∗ Charlson comorbidity index is a measure of overall health.

### Gold Standard Assessments

The prediction models were built using a randomly selected sample of 900 Michigan patients. Eligible patients were aged 40 to 80 years with ≥2 visits to eye care professionals. Two ophthalmologists (J.M.R. and A.D.Z.) each evaluated the EHRs of the 900 patients and categorized each eye of each patient as having definite, possible, or no evidence of glaucoma, DR, and AMD. A third ophthalmologist (A.K.B.) served as an adjudicator if determinations differed between the primary evaluators. The ophthalmologists could review any EHR area to make their diagnostic assessment except scanned documents in the “media” tab and ocular diagnostic tests from the Picture Archiving and Communication System. Some patients had only 2 visit encounters; others had many. Based on these ratings, a *definite glaucoma* variable (yes/no) was used as the gold standard for building the EPI prediction model. Variables for *definite DR* and *definite AMD* were created similarly. “Possible glaucoma” was applied when EHR evidence of the diagnosis was inconclusive, including glaucoma suspects or patients with ocular hypertension. For all 3 models, persons considered possible case patients were treated as noncases. To avoid bias, the 3 raters were masked to the overall project purpose and each other’s assessments.

### Creating Models to Predict Likelihood of Developing Eye Disease

Model building and calibration were performed using R software (version 4.3.1, including glmnet,^16^ caret,^17^ rms,^18^ R Foundation for Statistical Computing). Logistic least-absolute shrinkage and selection operator (LASSO) regression determined which EPI model covariates together best predicted the presence of glaucoma, DR, or AMD relative to the gold standard ophthalmologist gradings.^19^ Least-absolute shrinkage and selection operator constrains the magnitude of the regression coefficients, favoring models with fewer predictors, inducing variable selection in the fitting process. A benefit of LASSO for variable selection is its flexibility to include a variable yet penalize its coefficient when a strict inclusion or exclusion decision is too restrictive. Before the LASSO regression, categorical predictors with >99% of cases in a single category were excluded. Additional machine learning models, including random forest, were also tested and generated similar results to those of the LASSO regression. Patients were split 70% and 30% (630/270) into training and holdout sets. The optimality criterion was the 10-fold cross-validated mean prediction error in the training set. Global discrimination criteria (e.g., area under the receiver operating curve [AUC] and area under the precision–recall curve [AUPRC]) and local performance criteria dependent on the F1-optimal threshold (e.g., sensitivity, specificity, positive predictive value [PPV], and negative predictive value) were computed on the holdout set; standard errors were estimated with bootstrapping. International Classification of Diseases-only models, which predicted the presence of disease solely by the presence of ≥1 appropriate ICD code, were used as a baseline for comparison.

After we identified the LASSO model that best predicted which patients had glaucoma, DR, and AMD, we applied our EPI prediction model to eyes of all 319 562 Michigan patients in SOURCE, allowing us to estimate each eye’s probability (0%–100%) of having each condition. We compared demographics of patients with low (minimum) and high (top 5%) scores for each condition to assess whether they align with previously established demographic factors associated with these conditions.

### Model Calibration

We checked the calibration of each probability model. We used stratified sampling to identify Michigan patients whose probabilities of glaucoma (198 patients, 396 eyes), DR (196, 392), and AMD (197, 394) were uniformly distributed between 0 and 1.^20^ The ophthalmologists evaluated these patients’ EHRs as before. We used the Michigan calibration sets to compute calibration-in-the-large, calibration intercept and slope, and the maximum distance (Emax) between the loess calibration curve and ideal 45˚ line. We computed AUCs using inverse probability weighting (IPW) to account for the stratified sampling.^21^ This comprehensive approach ensures robust evaluation of the model's calibration and discrimination performance across a representative patient population.

### Identifying Clinical Features Contributing Most to the EPI Model Predictions

For each condition, we identified the top clinical features from our LASSO regressions. We also used random forest modeling to identify the clinical characteristics with the highest importance rankings.

### External Site Validation

We performed an external site validation of our models using data from Stanford University. Using similar methods as described earlier, 2 ophthalmologists at Stanford (C.L. and B.M.) reviewed the EHR records of 1057 randomly selected patients (2114 eyes) to determine the presence or absence of glaucoma, DR, and AMD. A third Stanford ophthalmologist (W.-S.L.) served as an adjudicator.

## Results

### Description of the University of Michigan Sample

The mean (± standard deviation) age of the 900 patients was 64 ± 12 years; the sample included 498 women (55%) and 714 White (79%), 86 Black (10%), 46 Asian American (5%), and 29 Latinx (3%) patients. Based on the graders’ assessments, 160 eyes had glaucoma (8.9%), 138 had DR (7.7%), and 106 had AMD (5.9%) (Table S3, available at www.ophthalmologyscience.org). This sample was split into a training set (n = 630) and a holdout set (n = 270).

### Model Performance in Predicting Disease Presence

In the training set, the EPI models developed for glaucoma, DR, and AMD performed better on most metrics (Table S4, available at www.ophthalmologyscience.org**)** including model accuracy, specificity, PPV, and F1 score. When applied to the holdout set at Michigan, the EPI models outperformed the ICD-only models for each of the 3 conditions. The EPI model for glaucoma had an AUC (± standard error) of 0.97 ± 0.008 and an AUPRC of 0.79 ± 0.04, whereas the ICD-only model had 0.90 ± 0.014 for AUC and 0.32 ± 0.034 for AUPRC. For DR, in the EPI model, AUC was 0.997 ± 0.001 and the AUPRC 0.96 ± 0.02; in the ICD-only model, AUC was 0.98 ± 0.010 and AUPRC 0.84 ± 0.042. For AMD, in the EPI model, AUC was 0.99 ± 0.004 and the AUPRC 0.74 ± 0.065; in the ICD-only model, AUC was 0.95 ± 0.018 and AUPRC 0.55 ± 0.056 (Table 5). The accuracy for glaucoma, DR, and AMD was 0.95, 0.98, and 0.97 for the EPI models and 0.85, 0.99, and 0.96 for the ICD-only models, respectively. For all 3 diseases, specificity and PPV were better with the EPI models than with the ICD-only models (Table 6).

**Table 5:** Comparison of the Area Under the Receiver Operating Curve and Area Under the Precision–Recall Curves for the Enhanced Phenotype Identification Models with the ICD-Only Models for Glaucoma, Diabetic Retinopathy, and Macular Degeneration

Condition	Method-Site	AUC, Mean (SE)	AUPRC, Mean (SE)
Glaucoma	ICD-UM	0.90 (0.014)	0.32 (0.034)
	EPI-UM	0.97 (0.008)	0.79 (0.040)
	ICD-SF	0.91 (0.005)	0.64 (0.014)
	EPI-SF	0.93 (0.005)	0.74 (0.016)
DR	ICD-UM	0.98 (0.010)	0.84 (0.042)
	EPI-UM	0.997 (0.001)	0.96 (0.020)
	ICD-SF	0.97 (0.008)	0.77 (0.027)
	EPI-SF	0.98 (0.005)	0.77 (0.033)
AMD	ICD-UM	0.95 (0.018)	0.55 (0.056)
	EPI-UM	0.99 (0.004)	0.74 (0.065)
	ICD-SF	0.93 (0.006)	0.53 (0.019)
	EPI-SF	0.96 (0.005)	0.64 (0.027)

AMD = age-related macular degeneration; AUC = area under the receiver operating curve; AUPRC = area under the precision recall curve; DR = diabetic retinopathy; EPI = enhanced phenotype identification; ICD = International Classification of Diseases; SE = standard error; SF = Stanford University; UM = University of Michigan (holdout set).

**Table 6:** Comparison of Several Common Performance Metrics for the Enhanced Phenotype Identification Models with the ICD-Only Models for Glaucoma, Diabetic Retinopathy, and Macular Degeneration

Disease	Model-Site	Accuracy	Sensitivity	Specificity	PPV	NPV	F1
Glaucoma	ICD-UM	0.85 (0.012)	0.95 (0.026)	0.84 (0.013)	0.33 (0.033)	0.995 (0.003)	0.49 (0.037)
	EPI-UM	0.95 (0.007)	0.72 (0.054)	0.97 (0.006)	0.64 (0.055)	0.98 (0.005)	0.67 (0.045)
	ICD-SF	0.88 (0.005)	0.96 (0.008)	0.86 (0.006)	0.65 (0.014)	0.99 (0.002)	0.77 (0.010)
	EPI-SF	0.87 (0.005)	0.49 (0.017)	0.97 (0.003)	0.84 (0.017)	0.87 (0.006)	0.62 (0.016)
DR	ICD-UM	0.99 (0.004)	0.98 (0.019)	0.99 (0.004)	0.85 (0.040)	0.998 (0.002)	0.91 (0.025)
	EPI-UM	0.98 (0.005)	0.84 (0.045)	0.99 (0.003)	0.92 (0.034)	0.99 (0.004)	0.87 (0.030)
	ICD-SF	0.98 (0.002)	0.95 (0.016)	0.99 (0.002)	0.80 (0.024)	0.997 (0.001)	0.87 (0.016)
	EPI-SF	0.97 (0.003)	0.50 (0.036)	0.99 (0.001)	0.84 (0.033)	0.97 (0.003)	0.63 (0.032)
AMD	ICD-UM	0.96 (0.007)	0.94 (0.035)	0.96 (0.007)	0.58 (0.053)	0.996 (0.002)	0.72 (0.044)
	EPI-UM	0.97 (0.006)	0.84 (0.051)	0.98 (0.005)	0.71 (0.056)	0.99 (0.003)	0.77 (0.043)
	ICD-SF	0.91 (0.005)	0.96 (0.010)	0.91 (0.005)	0.55 (0.019)	0.994 (0.001)	0.70 (0.016)
	EPI-SF	0.92 (0.005)	0.49 (0.026)	0.97 (0.003)	0.67 (0.028)	0.94 (0.004)	0.57 (0.023)

AMD = age-related macular degeneration; DR = diabetic retinopathy; EPI = enhanced phenotype identification; F1 = F1 score; ICD = International Classification of Diseases; NPV = negative predictive value; PPV = positive predictive value; SF = Stanford University; UM = University of Michigan (holdout set).

Values are reported as means with standard errors.

### Model Calibration

The glaucoma model demonstrated calibration-in-the-large, as the mean predicted value (0.48) and the proportion of glaucoma cases (0.53) were similar. The calibration intercept and slope of the glaucoma model were 0.31 and 1.25, close to their ideal values of 0 and 1, respectively. The slope was not <1, which would have indicated an overfit model. Emax was 0.15, indicating good calibration. The IPW-estimated AUC was 0.969. The EPI model for DR demonstrated calibration-in-the-large, as the mean predicted value (0.53) and the proportion of DR cases (0.54) were close. The calibration intercept and DR model slope were 0.19 and 1.04, close to their ideal values (0 and 1). Emax was 0.06, indicating very good calibration. The IPW-estimated AUC was 0.984. The AMD model underpredicts disease (calibration-in-the-large). For example, the mean predicted value (0.45) is lower than the proportion of AMD cases (0.56). The direction of the miscalibration of all the statistics is consistent with underpredicting the probability of disease. This may result from very few patients with AMD in training the model. The model did not assign a high enough probability because it lacked sufficient cases for high certainty. The maximum AMD probability among Michigan patients was 0.73; however, the IPW-estimated AUC, 0.993, indicates outstanding discrimination (Fig S1, available at www.ophthalmologyscience.org).

### Most Important Disease Prediction Variables

In the EPI glaucoma model, 11 variables, including 4 optic nerve descriptors, best predicted together whether a patient had glaucoma. The first and fourth most predictive variables were any record of a glaucoma medication prescription and ICD billing codes, respectively. The EPI model for DR comprised 7 variables, 4 of them involving macula examination findings; ICD codes were most predictive. The EPI AMD model had 8 variables. Four involved macula examination findings; ICD codes were again most predictive. For all conditions, most LASSO variables were also found to be most important when using random-forest modeling (Table 7).

**Table 7:** Variables Determined to Be of Greatest Importance for the Enhanced Phenotype Identification Models for Glaucoma, Diabetic Retinopathy, and Age-Related Macular Degeneration

Glaucoma			Diabetic Retinopathy			Macular Degeneration		
Variables	Estimates	VIP	Variables	Estimates	VIP	Variables	Estimates	VIP
Record of glaucoma medication use	3.07	1	ICD codes for DR	4.71	1	ICD codes for AMD	3.35	1
Absence of optic nerve findings∗	0.88	3	Evidence of BDR in macula examination	1.69	2	“Drusen” in macula examination	0.81	2
“Glaucomatous” optic nerve	0.80	6	Hemorrhages noted in macula examination	0.96	6	“Normal” macula examination	−0.53	5
ICD codes for glaucoma	0.78	2	Scarring noted in macula examination	0.61	11	Macula examination with other descriptors of AMD	0.48	4
“Normal” optic nerve	−0.71	5	Orders for intravitreal injections	0.56	8	Occupation: Retired	0.33	12
Pachymetry order	0.58	4	Exudates noted in macula examination	0.20	10	“Irregular” iris	0.24	51
+ RAPD	0.22	13	Systemic medication for diabetes	0.12	7	“RPE changes” in macula examination	0.21	8
Conjunctival injection	0.20	10				Marital status: Divorced	0.11	10
“Irregular” iris	0.18	52						
“Poor view” optic nerve	0.01	14						
Surgery to iris	0.01	20						

AMD = age-related macular degeneration; BDR = background diabetic retinopathy; DR = diabetic retinopathy; ICD = International Classification of Diseases; RAPD = relative afferent pupillary defect; RPE = retinal pigment epithelium; VIP = variable importance from random forest.

Estimates were generated from least-absolute shrinkage and selection operator (LASSO) regression enhanced phenotype identification (EPI) modeling. The greater the magnitude of the estimate, the greater the importance of that particular variable in predicting the phenotype of interest. Positive scores indicate the presence of the variable increases the likelihood of a patient having the phenotype of interest, while negative scores indicate the presence of the variable decreases the likelihood of a patient having the phenotype of interest. Variable importance is generated from a random forest EPI model.

∗ Absence of optic nerve findings refers to mentions such as “no disc hemorrhages.”

### Patient Characteristics for Lowest/Highest Disease Probabilities

Compared with patients with the lowest predicted glaucoma probability (≤5%), Michigan patients with the top 5% predicted probability for glaucoma were older (mean age, 68 vs. 40 years) and more likely to be Black (16% vs. 10%) and male (48% vs. 44%). For DR also, the top 5% were older (65 vs. 42 years) and more likely to be Black (15% vs. 9%) and male (52% vs. 43%). Compared with the lowest 5% predicted probability for AMD, the top 5% was older (76 vs. 37 years) and more likely to be White (90% vs. 74%) and female (62% vs. 55%) (Table 8).

**Table 8:** Characteristics of Eyes with Low (Minimum) and High (Top 5%) Predicted Probability Scores for Glaucoma, Diabetic Retinopathy, and Age-Related Macular Degeneration Based on Our Enhanced Phenotype Identification Models

Effect	Category	Glaucoma		Diabetic Retinopathy		Macular Degeneration	
		Low Risk∗ (N: 362502)	High Risk (N: 34102)	Low Risk∗ (N: 529103)	High Risk (N: 31998)	Low Risk∗ (N: 415712)	High Risk (N: 33438)
Mean [SD] Age (yrs)		40 [25]	68 [16]	42 [25]	65 [15]	37 [23]	76 (11)
Sex, n (%)	Female	204264 (56)	17869 (52)	301066 (57)	15484 (48)	230677 (55)	20845 (62)
	Male	158238 (44)	16233 (48)	228037 (43)	16514 (52)	185035 (45)	12593 (38)
Race, n (%)	White	275007 (76)	25271 (74)	402727 (76)	24159 (76)	305523 (74)	30069 (90)
	Black	34852 (10)	5361 (16)	48270 (9)	4656 (15)	43276 (10)	977 (3)
	Asian American	27993 (8)	1676 (5)	42604 (8)	1207 (4)	37205 (9)	1080 (3)
	AI/others	24650 (7)	1794 (5)	35502 (7)	1976 (6)	29708 (7)	1312 (4)
Ethnicity, n (%)	Non-Hispanic	330333 (91)	30740 (90)	482128 (91)	29126 (91)	379144 (91)	30479 (91)
	Latinx	13864 (4)	808 (2)	18910 (4)	1025 (3)	16957 (4)	384 (1)
	Unknown/others	18305 (5)	2554 (8)	28065 (5)	1847 (6)	19611 (5)	2575 (8)

AI = American Indian; SD = standard deviation.

∗ The “low risk” group is everyone who has no risk factors. More than 5% (in fact, >50%) are tied with the lowest score. Thus, this group is much larger than the high-risk group, which is truly 5% of the total.

### External Site Validation

The Stanford sample comprised 2114 eyes of 1057 patients. The mean age was 72 ± 7.2 years; there were 618 female patients (59%) and 576 White (55%), 36 Black (3%), 230 Asian American (22%), and 101 Latinx (10%) patients. Based on graders’ assessments, 454 eyes (22%) had glaucoma, 126 eyes (6%) had DR, and 230 eyes (11%) had AMD (Table S3). When the Michigan-trained models were applied to the Stanford cohort, the EPI model outperformed the ICD-only model on AUC and AUPRC for glaucoma (0.93 vs. 0.91 and 0.74 vs. 0.64, respectively), DR (0.98 vs. 0.97 and 0.77 vs. 0.77, respectively), and AMD (0.96 vs. 0.93 and 0.64 vs. 0.53, respectively) (Table 5). Other model performance metrics varied depending on the disease (Table 6).

## Discussion

Using machine learning, we created EPI models to identify the presence or absence of glaucoma, DR, and AMD in the large real-world SOURCE data repository. Our approach incorporates various EHR fields for evidence of disease, moving beyond sole reliance on ICD billing codes. In comparing the performance of our EPI models to gold standard evaluations by board-certified ophthalmologists, our EPI models demonstrated superior performance relative to the ICD-only models. When tested on the holdout set at the University of Michigan, for all 3 conditions, our EPI models achieved accuracy levels ≥95%, AUCs ≥0.97, and AUPRCs ≥0.74. When the EPI models were tested on data from a second SOURCE site, they continued to perform well, with AUCs >0.93.

Although the models relying exclusively on ICD codes performed modestly well for all 3 conditions on accuracy (85%–99%) and AUC (90%–98%), the EPI models performed better on PPV and AUPRC. A higher PPV means that patients flagged as having disease by the EPI models are considerably more likely than those identified using ICD-only models to actually have the condition. The AUPRC can be useful with imbalanced data (considerably more cases than noncases or vice versa) and when finding positive examples is a priority. Although glaucoma, DR, and AMD are among the most common causes of vision loss nationally, their prevalences are likely to still be relatively low in large datasets of patient populations in private practice or tertiary care settings. The higher AUPRCs indicate that patients with these conditions are more likely to be accurately identified using our EPI models, compared with ICD-only models. The importance of these performance metrics can vary depending on the study investigators’ specific research question.

Using EPI models has additional advantages. With ICD-only models, each patient in a given dataset either has documentation of encounters with the ICD code of interest or not—a binary yes/no determination. With EPI models, each patient is given a probability, ranging from 0 to 1, of having the disease. In turn, the researcher has more flexibility in deciding which patients to count as having the disease, depending on the study. For selected studies, the probability threshold for having the disease may be set high; the sample size of patients with the diagnosis is relatively small, but researchers can be highly confident of the patients’ diagnosis. For studies with a lower selected threshold, the sample size of eligible patients would increase, but a subset of patients may be misclassified with the disease. The added flexibility afforded by EPI models may be particularly useful for identifying potential patients from large datasets for clinical trial recruitment.^22^ Additionally, EPI models could address biases by identifying more patients who may be missing information in their EHRs due to systematic biases, thereby improving the inclusivity and accuracy of the dataset.

The larger goal of this research is not only to use EPI modeling to identify the presence or absence of ocular diseases but also to leverage this approach to characterize eyes with different ocular manifestations or severities of the diseases of interest. For example, among eyes that have been identified with glaucoma, additional EPI algorithms can distinguish glaucoma suspects from those with bona fide disease and patients with mild disease from others with severe disease or slow progressors from rapid progressors. Likewise, for patients identified with DR, additional EPI algorithms can distinguish eyes with nonproliferative DR from others with proliferative DR or nonexudative AMD from others with exudative AMD. Researchers can also leverage our algorithms to identify subsets of patients who experience progression from a less severe to a more severe disease state. In recent years, several genome-wide association studies have sought to identify all the genes associated with an ocular phenotype of interest.^23^^,^^24^ We hope these EPI models will help better identify and characterize ocular phenotypes, allowing for phenome-wide association studies in ophthalmology.^25^

While we could have used findings from ocular diagnostic tests such as visual fields or OCT to determine which patients did and did not have these 3 common eye diseases of interest for model training and validation, we opted not to do so because only a subset of patients receive all these ocular diagnostic tests as part of routine care, and we wanted a system that could generate predictions for all adults in a given dataset. While there were a few fringe cases that the board-certified ophthalmologists at the 2 sites who were tasked to review the patient records could not agree with one another for disease presence or absence, the level of agreement among the 2 primary reviewers was 92%, 99%, and 97% for glaucoma, DR, and AMD, respectively.

We know of a few other studies that sought to develop more detailed means of identifying patients with common ocular phenotypes beyond exclusive ICD code reliance. Using Veterans Administration data, Biggerstaff et al developed algorithms for detecting patients with primary open-angle glaucoma.^26^ Their best-performing algorithm involved ≥2 ICD billing codes for the condition plus glaucoma medication prescriptions. Their algorithm's PPV, 76.3%, was slightly higher than our Michigan-EPI glaucoma model’s; however, their negative predictive value, 88.2%, was lower than ours. Similar to that study, our EPI model included ICD codes and medication prescriptions but also included clinical examination findings (e.g., descriptions of optic nerve, conjunctiva, and pupils), perhaps accounting for our improved PPV. Restrepo et al created an algorithm for glaucoma detection in Black patients at Vanderbilt University^27^ using ICD-9 codes, Current Procedural Terminology codes, and free-text data; their PPV was 51.6%. Using Veterans Administration data, an algorithm developed by Nealon et al to identify primary open-angle glaucoma patients/nonpatients achieved a PPV of 83.5% and a negative predictive value of 97.5%.^28^ Their final algorithm required 6 eye-care visits with ICD codes for glaucoma and ≥2 intraocular pressure lowering drug prescriptions. That study’s PPV was higher than ours; however, applying their models in future research could be somewhat limiting, as patient eligibility would require sufficient eye-care visits, or if they receive only once-yearly care, require 6 years in the health care system. Direct comparisons between these studies and ours are challenging, partly because they aimed to identify patients with primary open-angle glaucoma, whereas we considered any glaucoma type.

Another study, by Restrepo et al,^29^ used EHR data to develop algorithms to identify patients with DR among Black persons with type 2 diabetes mellitus seen at Vanderbilt University. Their algorithm used ICD-9 codes, Current Procedural Terminology codes for therapeutic DR interventions, medications, and free-text data mining. The model’s accuracy, 84.8%, and PPV, 75.3%, were lower than ours; however, direct comparison of the studies is limited by the different study populations and other factors.

Our study has several limitations. The data to train and validate our EPI models involved patients from large tertiary-care health systems. Michigan and Stanford patients may be more clinically complex and sociodemographically dissimilar to patients in other care settings. Our EPI models would benefit from further validation using patient data from additional settings, including private practices or the Veterans Administration. Although all of the model training and validation used data from patients receiving care at institutions on EPIC, there is no reason our algorithms should not work on other EHR systems so long as they capture all of the variables deemed to be of most importance in Table 7. The level of detail documented at eye care visits may vary depending on factors like the practice setting, the clinical volume, the EHR software, and the presence of trainees or medical scribes. Second, our model would be affected if a patient was misdiagnosed or the presence or absence of observed disease features that our EPI models associate with disease presence or absence went undocumented. A third limitation of this study is our decision to require ≥2 visits as one of the study inclusion criteria. We did this to permit us in subsequent research to assess for changes in the status of one of these diseases of interest over time. However, requiring ≥2 visits may bias our study sample toward those with better access to eye care services. Fourth, while a main goal of our EPI modeling is to maximize predictive accuracy and a side benefit is a reduced list of variables needed to make predictions, it is important to acknowledge the resulting variable set should not be interpreted causally, and occasionally unusual variables (e.g., divorced status in the AMD model) are selected from the many available correlated variables. Finally, free text and ocular diagnostic test data are not yet incorporated into our algorithms. Such model inputs, planned for future iterations, will hopefully improve on the already strong performance of our EPI algorithms.

While sole reliance on ICD billing codes may be sufficient to answer certain research questions regarding ocular diseases, the use of EPI algorithms, including the ones we present, should greatly expand the depth and breadth of questions researchers can answer using real-world ophthalmology data. Enhanced phenotype identification algorithms allow for more accurate and nuanced identification of disease phenotypes, providing researchers with the flexibility to enhance the potential for robust and comprehensive analysis, addressing systematic biases, and improving patient inclusivity in clinical studies. Enhanced phenotype identification algorithms can profoundly impact the depth and breadth of ophthalmology research, leading to better-informed clinical decisions and improved patient outcomes.
