# Supplementary material for: Enhanced Phenotype Identification of Common Ocular Diseases in Real-World Datasets
Source: Ophthalmol Sci. 2025 Jan 24;5(4):100717. doi: 10.1016/j.xops.2025.100717 (PMC11985028; doi:10.1016/j.xops.2025.100717)
Supplement: Figure S1 [file mmc1.pdf]

Figure S1. Calibration Plots for the Enhanced Phenotype Identification Prediction Models for Glaucoma (left), Diabetic Retinopathy (center), and Age-related Macular Degeneration (right).

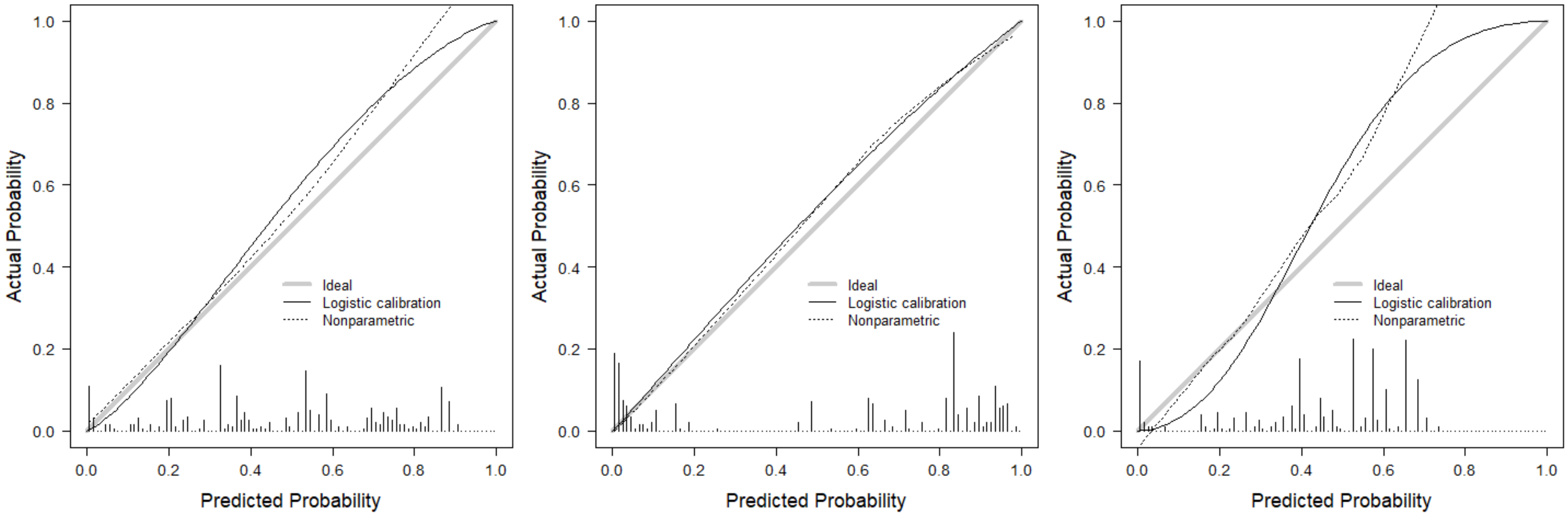

Grey curve is ideal 45° line representing equality of predicted probability (x-axis) and observed proportion (y-axis). Solid curve is from a parametric calibration curve; dashed line is from a loess local polynomial smoother. The three curves will be close to each other for a well-calibrated model.
