# Supplementary material for: Enhanced Phenotype Identification of Common Ocular Diseases in Real-World Datasets
Source: Ophthalmol Sci. 2025 Jan 24;5(4):100717. doi: 10.1016/j.xops.2025.100717 (PMC11985028; doi:10.1016/j.xops.2025.100717)
Supplement: Table S2 [file mmc2.pdf]

Table S2: Regular Expressions Used to Identify Key Clinical Exam Findings

<table>
<tr><th>Variable</th><th>Exam</th><th>Model(s)</th><th>Regexs</th></tr>
<tr><td>no disc heme</td><td>Optic Nerve</td><td>GLA</td><td>\bno (dh|(disc)?hem(e|orrhage)?|nvd?|(optic nerve)?edema|neovasc(ularization)?|(disc|waxy)? pallor|(disc)?swelling|notch|hypoplasia)\b</td></tr>
<tr><td rowspan="6">glaucomatous</td><td rowspan="6">Optic Nerve</td><td rowspan="6">GLA</td><td>glaucomatous|erosion|excavated</td></tr>
<tr><td>(cupp(ed|ing)|cup to (inf|sup)|\bntc\b)</td></tr>
<tr><td>(deep|total|large) cup</td></tr>
<tr><td>(enlarged|increased) c/d</td></tr>
<tr><td>\b(thin(ning|ner)?|notch?|rim|loss)\b</td></tr>
<tr><td>(inf(erior)?|sup(erior)?|(infero)?temp(oral)?|nas(al)?|st|it|sn|in|pole|rim)</td></tr>
<tr><td>normal*</td><td>Optic Nerve</td><td>GLA</td><td>\b(norma?l?|w?nl|healthy|flat|pink|sharp|(full|good|intact|uniform) rims?|rims? intact)\b</td></tr>
<tr><td>poor view</td><td>Optic Nerve</td><td>GLA</td><td>\b(no(clear)?|hazy|miotic|poor|difficult|limited|blurry|minimal|hard|difficult|fleeting|quick|unclear) (view|exam|glimpse))\b
(unable to see|not visible|no details?)</td></tr>
<tr><td rowspan="2">rapd</td><td rowspan="2">Pupil</td><td rowspan="2">GLA</td><td>(r?apd|\+|\btrace\b|\bslight\b|[1-9]|pos)</td></tr>
<tr><td>/b(yes|questionable|possible|reverse)/b</td></tr>
<tr><td>Irregular</td><td>Iris</td><td>GLA, AMD</td><td>\b(irreg(ular(ly)?)?|surgical|eccentric|oblong|peak|distorted|fixed)\b</td></tr>
<tr><td>bdr</td><td>Macula</td><td>DR</td><td>\b(bdr|ma'?s?\b|micro[-]an|dot blot)</td></tr>
<tr><td rowspan="2">hemorrhage**</td><td rowspan="2">Macula</td><td rowspan="2">DR</td><td>\b(heme?|dbh)\b</td></tr>
<tr><td>\bhem[morhage]*\b</td></tr>
<tr><td>scarring</td><td>Macula</td><td>DR</td><td>\bmac(ular)?[-]s[ct]ar</td></tr>
<tr><td>exudate</td><td>Macula</td><td>DR</td><td>\bexudate\b</td></tr>
<tr><td>drusen</td><td>Macula</td><td>AMD</td><td>\bdru[sz]e?n?\b</td></tr>
<tr><td>normal*</td><td>Macula</td><td>AMD</td><td>\b(norma?l?|good|flat)\b</td></tr>
<tr><td>macular degeneration</td><td>Macula</td><td>AMD</td><td>\bmacular degen(eration)?\b</td></tr>
<tr><td>rpe</td><td>Macula</td><td>AMD</td><td>\b(rpe|pigment(ary)?)(/b|(mot|elev|atr|thin|drop|clump|hyper|pig|ch))</td></tr>
<tr><td></td><td></td><td></td><td></td></tr>
<tr><td colspan="2">*overruled by any other pathology</td><td></td><td></td></tr>
<tr><td colspan="2">**Also 1 when BDR = 1</td><td></td><td></td></tr>
<tr><td colspan="2">Regex notes
all matching done case insensitive
whitespace collapsed prior to search
\b indicates start or end of word
? Indicates optional match (0 or 1 times)
* indicates optional match (0 or more times)
+ indicates match (1 or more times)
[] indicates match set
() indicates to consider contents as a single entity
| indicates options within ()</td><td></td><td></td></tr>
</table>
