# Supplementary material for: Enhanced Phenotype Identification of Common Ocular Diseases in Real-World Datasets
Source: Ophthalmol Sci. 2025 Jan 24;5(4):100717. doi: 10.1016/j.xops.2025.100717 (PMC11985028; doi:10.1016/j.xops.2025.100717)
Supplement: Table S3 [file mmc3.pdf]

Table S3. Characteristics of Patients in the University of Michigan Set and Stanford University (External Validation) Set

		University of Michigan*	Stanford University: External Validation Set
Number of Patients		900	1057
Number of Eyes		1800	2114
Sex	Female	498 (55.3)	618 (58.5)
	Male	402 (44.7)	439 (41.5)
Race	White	714 (79.3)	576 (54.5)
	Black	86 (9.6)	36 (3.4)
	Asian American	46 (5.1)	230 (21.8)
	AI/Others	54 (6.0)	215 (20.3)
Ethnicity	Non-Hispanic	811 (90.1)	919 (86.9)
	Latinx	29 (3.2)	101 (9.6)
	Unknown	60 (6.7)	37 (3.5)
Marital Status	Married	517 (57.4)	683 (64.6)
	Single	177 (19.7)	142 (13.4)
	Divorced	73 (8.1)	207 (19.6)
	Others	133 (14.8)	25 (2.4)
CCI, mean [SD]		3.6 [2.7]	3.2 [3.1]
Age at most recent visit (years), mean [SD]		64 [12]	72 [7.2]
Eyes with Glaucoma		160 (8.9)	454 (21.5)
Eyes with AMD		106 (5.9)	230 (10.9)
Eyes with DR		138 (7.7)	126 (6.0)

AI = American Indian; AMD = Age-related macular degeneration; CCI=Charlson comorbidity index; DR = Diabetic retinopathy; SD = standard deviation

*This includes the training set (n=630) and holdout set (n=270)
