# Supplementary material for: Enhanced Phenotype Identification of Common Ocular Diseases in Real-World Datasets
Source: Ophthalmol Sci. 2025 Jan 24;5(4):100717. doi: 10.1016/j.xops.2025.100717 (PMC11985028; doi:10.1016/j.xops.2025.100717)
Supplement: Table S4 [file mmc4.pdf]

Table S4. Model performance Metrics for the Enhanced Phenotype Identification Models with the ICD-Only Models for Glaucoma, Diabetic Retinopathy, and Macular Degeneration Among Patients in the University of Michigan Training Set

Disease	Model-Site	Accuracy	Sensitivity	Specificity	PPV	NPV	F1
Glaucoma	ICD-UM	0.83	0.98	0.82	0.36	0.997	0.52
	EPI-UM	0.97	0.90	0.98	0.79	0.99	0.834
DR	ICD-UM	0.98	0.94	0.99	0.83	0.995	0.88
	EPI-UM	0.99	0.87	0.997	0.95	0.99	0.91
AMD	ICD-UM	0.94	0.96	0.94	0.50	0.997	0.66
	EPI-UM	0.97	0.88	0.98	0.71	0.99	0.79

AMD = Age-related macular degeneration; DR = Diabetic retinopathy; F1 = F1 Score; NPV = Negative predictive value; PPV = Positive predictive value
